# The CMV early enhancer/chicken β actin (CAG) promoter can be used to drive transgene expression during the differentiation of murine embryonic stem cells into vascular progenitors

**DOI:** 10.1186/1471-2121-9-2

**Published:** 2008-01-11

**Authors:** Annika N Alexopoulou, John R Couchman, James R Whiteford

**Affiliations:** 1 National Heart and Lung Institute, Sir Alexander Fleming Building, Faculty of Medicine, Imperial College London SW7 2AZ, UK; 2 MRC Prion Unit, Institute of Neurology, UCL, Queen Square, London WC1N 3BG, UK; 3 Biomedicinsk Institute, University of Copenhagen, Biocenter, Ole Maoløes Vej 5, 2200 Copenhagen N, Denmark

## Abstract

**Background:**

Mouse embryonic stem cells cultured *in vitro *have the ability to differentiate into cells of the three germ layers as well as germ cells. The differentiation mimics early developmental events, including vasculogenesis and early angiogenesis and several differentiation systems are being used to identify factors that are important during the formation of the vascular system. Embryonic stem cells are difficult to transfect, while downregulation of promoter activity upon selection of stable transfectants has been reported, rendering the study of proteins by overexpression difficult.

**Results:**

CCE mouse embryonic stem cells were differentiated on collagen type IV for 4–5 days, Flk1^+ ^mesodermal cells were sorted and replated either on collagen type IV in the presence of VEGFA to give rise to endothelial cells and smooth muscle cells or in collagen type I gels for the formation of vascular tubes. The activity of the CMV and β-actin promoters was downregulated during selection of stable transfectants and during differentiation to the Flk1 stage, while the CMV immediate enhancer/β-actin promoter in the pCAGIPuro-GFP vector led to 100% of stably transfected undifferentiated and differentiated cells expressing GFP. To further test this system we expressed syndecan-2 and -4 in these cells and demonstrated high levels of transgene expression in both undifferentiated cells and cells differentiated to the Flk1 stage.

**Conclusion:**

Vectors containing the CAG promoter offer a valuable tool for the long term expression of transgenes during stem cell differentiation towards mesoderm, while the CMV and β-actin promoters lead to very poor transgene expression during this process.

## Background

Mouse embryonic stem (ES) cells are derived from the inner cell mass (ICM) of the mouse blastocyst and retain pluripotency when cultured *in vitro *in the presence of factors that inhibit differentiation, such as leukaemia inhibitory factor (LIF). However, when these factors are withdrawn, ES cells have the potential to differentiate into ectodermal, mesodermal, endodermal and germ cell lineages (reviewed in [1]). ES cells can differentiate either in suspension, forming embryoid bodies (EBs) or as two-dimensional adhesion cultures, either in the presence of feeder cells or extracellular matrix (ECM) molecules [1]. The regulation of ES cell differentiation is currently under intense study, not least for potential therapeutic use. One important area of investigation is ES cell differentiation either in EBs or in 2-dimensional culture, along pathways recapitulating vasculogenesis and early angiogenesis [2,3]. Related to this is delineation of functions for growth factors, adhesion molecules and transcription factors in the formation of the vasculature.

Elucidating the role of angiogenic factors during differentiation often requires over-expression of wild type and mutant proteins. Transfection of ES cells with plasmids using traditional electroporation and lipofectamine-based methods can be problematic [4-6]. Conflicting data about the activity of different promoters in undifferentiated ES cells have also been obtained, while several studies have suggested that transcriptional inactivation of promoters occurs during selection of stably transfected cells [7-9]. Another problem is obtaining stable transgene expression through stem cell differentiation. Several studies have looked at the efficacy of promoters in plasmid based vector systems with a view to obtaining high transient and stable expression in stem cells. For example, the CAG promoter has been used to express transgenes in undifferentiated ES cells as well as ES cells undergoing differentiation towards neuronal, myogenic and mesodermal cell types [10-12]. In the latter case a transfection system was used (ES cells expressing polyoma large T antigen and a vector with a polyoma virus origin of replication) leading to episomal propagation of the vector carrying the transgene of interest [12]. Although episomal plasmid propagation may lead to very high levels of transgene expression due to a high copy number of plasmid per cell, certain experiments may require lower levels of overexpression that may be more physiologically relevant. Here, we compare the promoter activity of the CMV, chicken β-actin and CAG promoters in undifferentiated murine embryonic CCE cells as well as during differentiation to mesoderm. These cells are routinely used for differentiation into mesodermal lineages [13] and do not express the polyoma virus large T antigen allowing integration of transgenes into the host's genome. We demonstrate that of these vector systems, only plasmids containing the CAG promoter can be used for long term expression of genes during differentiation of two dimensional cultures of mouse embryonic CCE ES cells grown on collagen substrates towards mesoderm.

## Results and Discussion

### Differentiation of mesodermal cell lineages from mouse CCE ES cells

**Figure 1:**
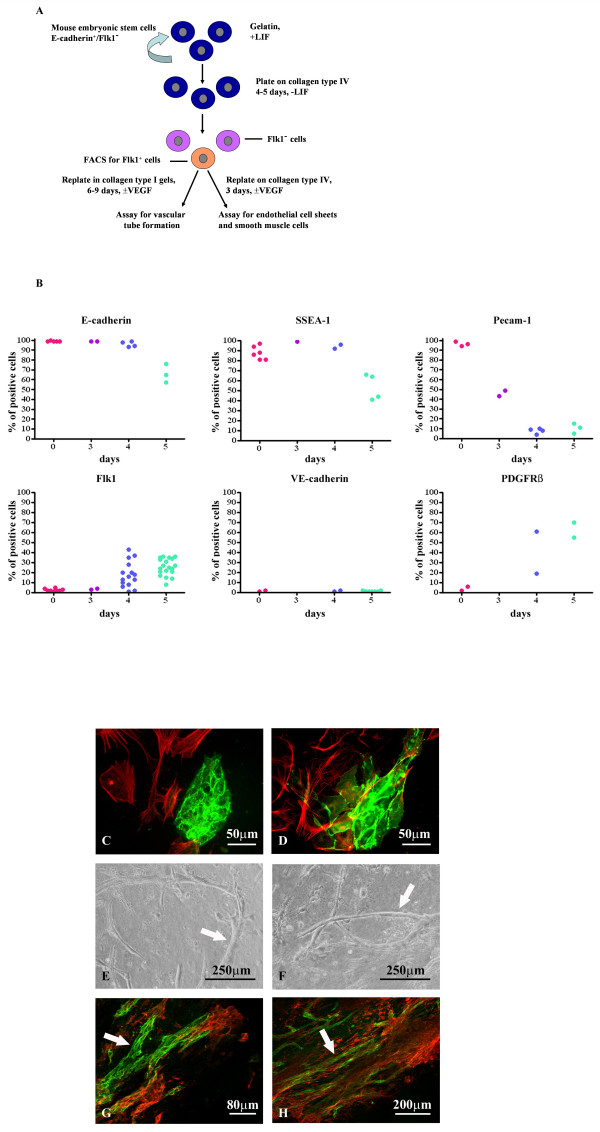
**ES cell differentiation into Flk1^+ ^mesodermal, endothelial and smooth muscle cells**. A. Schematic showing the protocol for differentiation of CCE cells to mesodermal lineages. B. Expression of cell surface markers at day 0 and days 3–5 of differentiation; cells were analysed by FACS for the markers shown. C-D. Flk1^+ ^cells were plated on collagen type IV in the presence of VEGFA. Cells were double immunostained for VE-cadherin (green) and αSMA (red) (C) or PECAM-1 (green) and αSMA (red) (D). EC sheets were found surrounded by SMCs. E-F. Phase contrast micrographs of Flk1^+ ^cells after FACS sorting and seeding into collagen type I gels in the presence of VEGFA for 6 days. Vascular tube structures are arrowed. G-H. Collagen I gels were sectioned and stained for Pecam-1 (green) and αSMA (red) and showed Pecam-1^+ ^tubular structures (arrows) surrounded by smooth muscle cells.

Cells of a mesodermal/endothelial lineage were differentiated from mouse CCE embryonic stem cells following the protocol described in [13] (Fig. 1A). Undifferentiated CCE cells expressed high levels of E-cadherin, SSEA-1 and Pecam-1, which is consistent with previous data [14-16]. These cells did not express the mesodermal/endothelial marker Flk1, the endothelial cell (EC) marker VE-cadherin or the smooth muscle cell (SMC)/pericyte marker PDGFRβ (Fig. 1B, day 0). Differentiation was initiated by the removal of LIF from the medium and the seeding of cells on collagen type IV. Flk1 expression was observed on days 4–5 of differentiation, while elevated levels of PDGFRβ were also noted at the same time. Expression of Pecam-1 was much reduced during differentiation and the levels of E-cadherin and SSEA-1 also declined by day 5. VE-cadherin levels remained negligible during this 5 day differentiation period (Fig. 1B).

Flk1^+ ^cells from either day 4 or day 5 cultures were FACS sorted and replated on collagen type IV-coated plates in the presence of VEGFA for a further 3 days (Fig. 1A). Double immunofluorescence microscopy for the EC markers VE-cadherin or Pecam-1 and the SMC marker α-smooth muscle actin (αSMA) (Fig. 1C, D) showed the presence of EC sheets surrounded by SMCs. Differentiation into ECs was VEGFA-dependent, as only αSMA^+ ^cells were observed in its absence (data not shown). When Flk1^+ ^cells were FACS sorted and plated in three dimensional collagen type I gels in the presence of VEGFA for an additional 6 days, the formation of angiogenic sprouts was observed (Fig. 1E, F). Tubular structures positive for the EC marker Pecam-1 were surrounded by αSMA^+ ^SMCs/pericytes (Fig. 1G, H). The formation of tubes was dependent on VEGFA/Flk1 signalling, since no tubes formed in the absence of VEGFA or when Flk1^- ^cells were sorted and cultured in the presence of VEGFA (data not shown). These data are in agreement with previous studies [13,3] and suggest that this differentiation protocol mimics aspects of both vasculogenesis and early angiogenesis and is suitable for ectopic expression studies.

### The CMV and β-actin promoters are not suitable for sustained expression of transgenes during CCE cell differentiation

**Figure 2:**
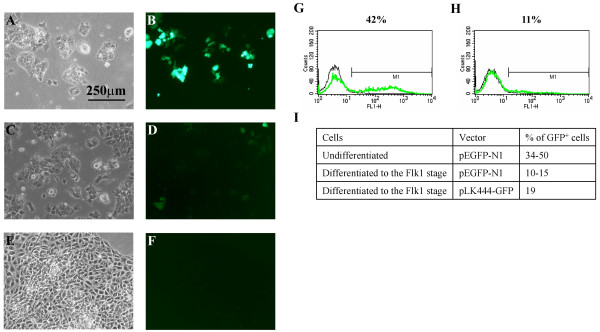
**The CMV promoter is down regulated in mouse embryonic CCE cells and during differentiation into mesoderm**. A-D. CCE cells were transfected with pEGFP-N1 and subjected to G418 selection 48 hours later. Cells expressing GFP are obvious 2 days after transfection (A, B) while few GFP positive cells remain 7 days post transfection (C, D). E-F. CCE cells were transfected with pEGFP-N1 and differentiation was initiated 2 days post transfection. No GFP^+ ^cells were observed on day 5 of differentiation to the Flk1 stage (pool of containing both Flk1+ and Flk1- cells) when transient transfectants were used. G-I. CCE cells were transfected with pEGFP-N1 and placed under G418 selection 2 days post transfection. Undifferentiated cells (G, I) and cells differentiated to the Flk1 stage (H, I) were analysed by FACS for GFP expression 14–19 days post transfection. The data suggest that less than 50% of the undifferentiated cells express GFP, while this percentage declines during differentiation to the Flk1 stage. Similar levels of GFP expression were obtained during differentiation of cells transfected with pLK444-GFP.

High levels of transfection efficiency (80% +) of CCE cells (Fig. 2A, B) were obtained with a modified lipofectamine based protocol where suspended cells were transfected with pEGFP-N1 [9]. This compared favourably with other methods such as electroporation (70% transfection efficiency; data not shown) and the standard lipofectamine protocol (40% efficiency; data not shown). However the longevity of GFP expression in transfected CCE cells was poor. After 7 days in culture under antibiotic selection very few fluorescing cells were evident (Fig. 2C, D) as compared to cultures 2 days after transfection (Fig. 2A, B). These results were confirmed by FACS analysis showing that only 34–50% of undifferentiated cells surviving antibiotic selection expressed GFP (Fig. 2G, I), while a dramatic decrease in the transgene's expression was noted in stably transfected cells differentiated for 5 days on collagen type IV to the Flk1 stage (Fig. 2H, I). It should be noted that at this stage the pool of cells contain both Flk1+ and Flk1- cells. The GFP cDNA in pEGFP-N1 is under the control of the CMV promoter and it is not uncommon for transcriptional inactivation of this promoter to occur in certain cell types. To overcome this problem we expressed GFP under the control of the chicken β-actin promoter. GFP expression in differentiated cells was again very low (Fig. 2I) and this promoter offered no advantage over the CMV promoter.

Since a higher percentage of transiently transfected cells expressed GFP compared to the stably transfected cells (Fig. 2A–D), cells 2 days post transfection were used for differentiation to test whether this would lead to more GFP^+ ^cells present at the Flk1 stage. However, the use of transiently transfected cells resulted in no cells expressing GFP on day 5 of differentiation (Fig. 2E, F). This suggested that stable integration of the transgene into the host's chromosome is required for its expression.

**Figure 3:**
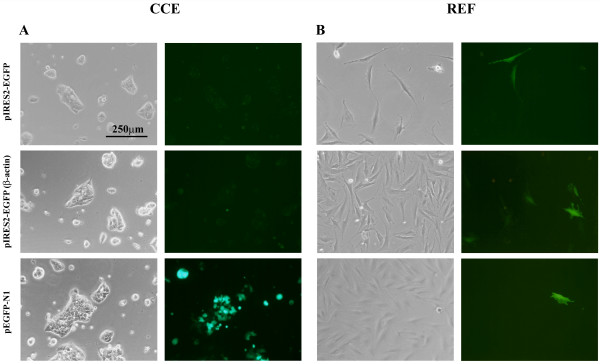
**The CMV and chicken β-actin promoters cannot drive gene expression in bicistronic vectors transfected into CCE cells**. CCE cells (A) or rat embryo fibroblasts (B) were transfected with the bicistronic vectors pIRES2-EGFP and pIRES-EGFP(β-actin) in which the CMV promoter had been substituted with the chicken β-actin promoter. GFP expression was not evident in CCE cells transfected with these vectors; GFP^+ ^cells were only obtained when cells were transfected with pEGFP-N1. In contrast GFP expression was apparent in fibroblasts transfected with all three constructs (B). Micrographs were obtained 24 hours after transfection.

From the above studies it was evident that resistance against antibiotic selection did not correlate with expression of the transgene of interest. Therefore, bicistronic vectors having GFP as the reporter gene were tested, as they would allow for easy identification and selection of cells expressing the transgene of interest. CCE cells were transfected with either the pIRES2-EGFP plasmid or pIRES2-EGFP(β-actin), where the CMV promoter had been substituted for the chicken β-actin promoter. However, GFP expression was not apparent in CCE cells transfected with either plasmid even 1 day after transfection (Figure 3A). Control cultures were transfected in parallel with pEGFP-N1 and GFP^+ ^cells were abundant in this case. The bicistronic vectors were then transfected into fibroblasts to check their functionality and fluorescing cells were obtained from transfections with both pIRES2-EGFP and pIRES2-EGFP(β-actin) (Fig. 3B).

The above data suggest that to obtain sustained transgene expression in murine CCE cells and during differentiation into mesoderm, alternative promoters to the CMV and the chicken β-actin promoters are required. Furthermore, the above promoters driving expression in a vector that contains an IRES element were inactive in undifferentiated ES cells, in agreement with previous reports [7].

### Vectors containing the CAG promoter can be used to express transgenes in murine CCE cells and during differentiation into mesoderm

The CMV immediate enhancer/β-actin (CAG) promoter has been shown to give higher levels of transgene expression in several cell lines compared to the CMV and β-actin promoters [17]. Two different vectors containing the CAG promoter were used. The first, pCAGIPuro is a bicistronic vector, where a puromycin resistance gene follows the IRES element. GFP was cloned into this vector to create pCAGIPuro-GFP (Table 1). The pCAGGS plasmid is a monocistronic vector with GFP expression also under the control of the CAG promoter. The G418 resistance cassette from vector pMC1neo polyA was cloned into pCAGGS to create pCAGGSneo-GFP (Table 1).

**Table 1:** Plasmids used in this study.

Plasmid	Promoter	Transgene/s	IRES element	Antibiotic resistance to
pEGFP-N1	CMV	GFP	No	G418
pIRES2-EGFP	CMV	GFP	Yes	G418
pIRES2(β-actin)	β-actin	GFP	Yes	G418
pLK444-GFP	β-actin	GFP	No	G418
pCAGIPuro-GFP	CAG	GFP and Puromycin resistance	Yes	Puromycin
pCAGGSneo-GFP	CAG	GFP	No	G418
pCAGIPuro-sdc2/4	CAG	Syndecan-2/4	Yes	Puromycin

**Figure 4:**
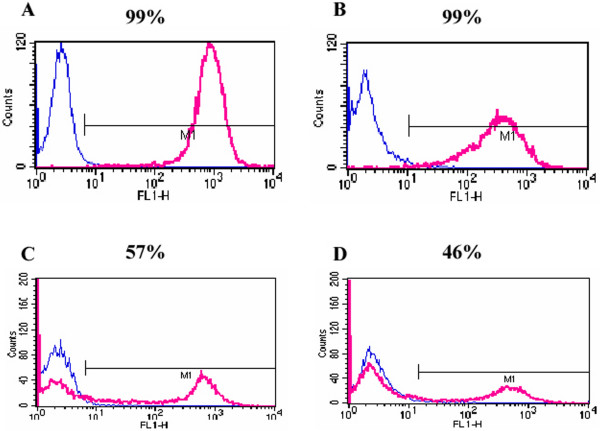
**Sustained GFP expression in CCE cells during differentiation into mesoderm is obtained using the CAG promoter**. CCE cells were transfected with pCAGIPuro-GFP (A, B) or pCAGGSneo-GFP (C, D) and selected for puromycin or G418 resistance, respectively. FACS analysis was performed on undifferentiated CCE cells (A, C) and cells differentiated to the Flk1 stage (B, D)14–18 days posts transfection and GFP expression was compared in vectors expressing GFP (red) and empty vector (blue) transfected controls.

CCE cells were transfected with pCAGIPuro-GFP and selected for puromycin resistance. FACS analysis for GFP expression showed that virtually 100% of either undifferentiated (Fig. 4A) or cells differentiated to the Flk1 stage (Fig. 4B) expressed high levels of GFP. Indeed, all of the cells in these differentiated cultures consisting of both Flk1+ and Flk1- cells were expressing GFP and this suggests that the CAG promoter is active in both Flk1+ and Flk1- cells. CCE cells were also transfected with the pCAGGSneo-GFP vector and were selected for G418 resistance. When stable transfectants were analysed by FACS, approximately 50% of either undifferentiated (Fig. 4C) or differentiated (Fig. 4D) cells were GFP^+^. While this was a substantially lower percentage of positive cells than that obtained with the pCAGIPuro-GFP vector, it was much superior to the pEGFP-N1 and pLK444-GFP vectors, particularly during differentiation (see Fig. 2I).

**Figure 5:**
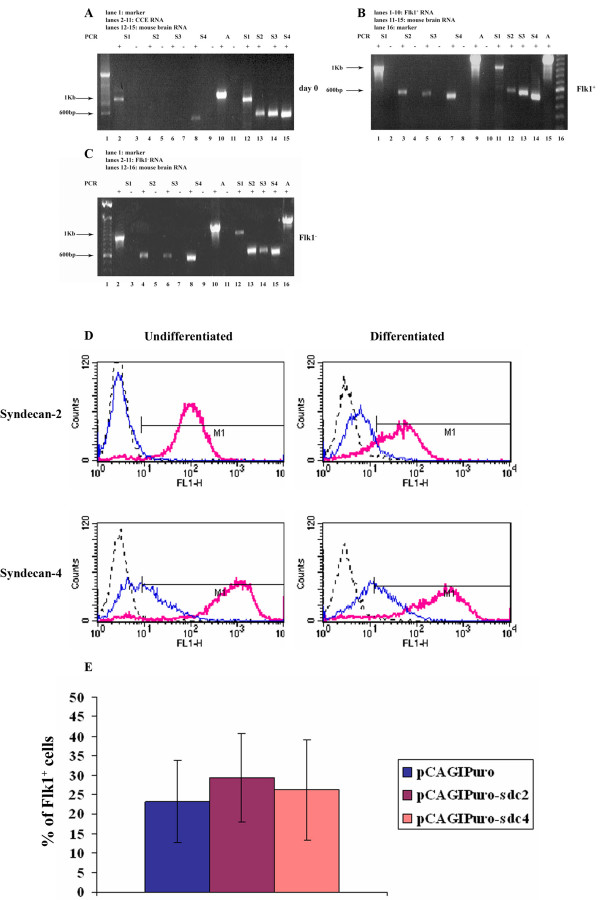
**Ectopic expression of Syndecan-2 and -4 through differentiation of CCE cells to mesodermal lineages**. Undifferentiated CCE cells (A) as well as Flk1^+ ^(B) and Flk1^- ^(C) cells FACS sorted on day 4 of differentiation were analysed by RT-PCR for syndecan expression. D. CCE cells were transfected with either pCAGIPuro-sdc2 or pCAGIPuro-sdc4 and were selected for puromycin resistance. FACS analysis was performed on undifferentiated and cells differentiated to the Flk1 stage as indicated using antibodies against syndecan-2 and -4. In each analysis, the dotted black line denotes the relevant secondary antibody control, the blue line corresponds to cells transfected with pCAGIPuro empty vector and the red line are cells transfected with constructs containing syndecan-2 or-4 cDNA. E. Levels of Flk1 were measured on day 5 of differentiation in cells transfected with pCAGIPuro-sdc2, pCAGIPuro-sdc4 or empty vector control. Data from 5 independent experiments are shown, where a total of 9 flasks were analysed for each construct. The bars represent the standard deviation, and the results were analysed with repeated-measures ANNOVA, using Prism Graphpad 4.0, assuming Gaussian distribution. No statistical significance was observed for the difference in FLK1 expression in cells transfected with the different constructs.

Having shown that the pCAGIPuro vector could be used to achieve sustained GFP expression during differentiation of CCE cells to mesodermal cell types we tested this vector system for its ability to express genes of biological interest. The syndecans are type I transmembrane proteoglycans involved in cell adhesion, migration and growth factor interactions [18]. In mammals there are four family members and we analysed undifferentiated and differentiating CCE cells for syndecan expression using RT-PCR. Undifferentiated CCE cells expressed only syndecans-1 and -4 (Fig. 5A), while Flk1^+ ^and Flk1^- ^cells FACS sorted on day 4 of differentiation expressed all four syndecans (Fig. 5B, C). We then decided to study syndecans-2 and -4 in further detail as they have been implicated in playing a role in the vascular system [19-23]. FACS analysis of undifferentiated CCE cells confirmed that cell surface syndecan-4 is present in these cells whereas syndecan-2 is not (Fig. 5D). It was only after differentiation that syndecan-2 appeared on the cell surface at low levels.

We then used the pCAGIPuro plasmid to express syndecans-2 and -4. Transfected cells were selected for puromycin resistance and the cells were differentiated on collagen type IV for 5 days. FACS analysis confirmed that high levels of cell surface expression of both proteins was achieved in undifferentiated as well as differentiated cells (Fig. 5D), although overexpression of these receptors had no effect on the percentage of Flk1^+ ^cells obtained during differentiation (Fig. 5E).

## Conclusion

Here we optimised a differentiation system of ES cells into Flk1^+ ^mesodermal cells, ECs and SMCs and subsequently used the cells to form angiogenic sprouts *in vitro*. We then evaluated a number of plasmid vector systems for expressing transgenes during differentiation to Flk1^+^mesodermal cells and found that vectors which utilise the CAG promoter are most effective at driving gene expression and do not compromise the ability of stem cells to differentiate. The CAG promoter provides long term expression of transgenes in these cells however the system is most effective when the cells can be put under a selective pressure to express the transgene. For this reason bicistronic vectors containing an antibiotic resistance gene offers the best way of obtaining expression of transgenes during differentiation into mesodermal Flk1+ cells. This is in contrast to vectors containing the CMV and β-actin promoters which gave very low levels of expression. This presents a very useful tool for use in studies of angiogenesis and vasculogenesis.

## Methods

### Cell lines and culture conditions

CCE mES cells (StemCell Technologies) were grown in flasks coated with 0.1% gelatin (StemCell Technologies) in Dulbecco's Modified Eagle Medium (DMEM) (StemCell Technologies) supplemented with 15% foetal calf serum (FCS) (StemCell Technologies), 100 μM 2-mercaptoethanol (Invitrogen), 10 ng/ml LIF (StemCell Technologies), 2 mM glutamine (Invitrogen) and 100 μM MEM non-essential amino acids (StemCell Technologies). The CCE cells were maintained in a humidified incubator at 37°C and 5% CO_2 _in air. Rat embryo fibroblasts (REFs) were cultured in alpha-Minimum Essential Medium (αMEM; Cambrex) supplemented with 5% FCS (Cambrex).

### Antibodies

The Flk1 (clone Avas12α; final concentration 50 μg/ml), the Pecam-1 (clone MEC13.3; 5 μg/ml) and the VE-cadherin (clone 11D4.1; 50 μg/ml) antibodies were from BD Pharmingen. The α smooth muscle actin (αSMA) antibody (1A4; 1 μg/ml) was from Sigma, the E-cadherin antibody (clone ECCD2; 20 μg/ml) was from Takara, Japan and the PDGFRβ antibody (10 μg/ml) was a kind gift from Dr W. Stallcup, Burnham Institute, La Jolla, California. R-Phycoerythrin-labelled SSEA-1 (clone 480; 4 μg/ml) and syndecan-2 antibodies (clone M140; 10 μg/ml) were from Santa Cruz. The polyclonal chicken anti-syndecan-4 antibody (Harlan Sera-Lab, UK) was raised against the N-terminal 20 amino acids of the mature human protein. Antibodies were affinity purified from plasma using standard procedures. The secondary antibodies (Molecular Probes) used were: Alexa-488 labelled donkey anti-rat IgG; Alexa-568 labelled goat anti-mouse IgG; Alexa-488 labelled goat anti-rabbit IgG; Alexa-488 labelled goat anti-chicken IgY, with working concentrations of 10 μg/ml.

### Flow cytometry analysis and sorting (FACS)

Cells were detached with cell dissociation buffer (Invitrogen) and stained in FACS buffer (0.5% BSA, 2 mM EDTA in 1× PBS) at 4°C. For exclusion of dead cells, the nucleic acid dye Topro-3 (0.1 μM; Molecular Probes) was used. The cells were analysed by a four colour FACS Calibur (BD) and data for 10,000 live cells were analysed by the CellQuest software. Cells stained with secondary antibody only or empty vector transfected cells served as controls for background fluorescence. The gate M1 was set to include 1% of the control cells that exhibited the highest fluorescence within the population and cells were considered to be positive when contained within the gate M1. For FACS sorting, dead cells were excluded by propidium iodide staining (0.5 μg/ml; Molecular Probes). Cell populations were sorted by FACS Diva (BD) and collected in sterile tubes in 2 ml medium. Flk1^+ ^cells were plated either on collagen type IV-coated plates or in collagen type I gels.

### Immunofluorescence microscopy

Cells on glass coverslips were fixed in acetone (-20°C) for 2 min. After washing in PBS, cultures were incubated with the appropriate concentration of primary antibody in PBS for 1 h at 37°C. Secondary antibody incubation for 1 h at 37°C was followed by further washing in PBS and coverslips were mounted with Immuno-Fluor (MP Biomedicals). Controls included staining with secondary antibody only, while in double labelling experiments, controls included incubation with one primary antibody and both secondary antibodies. Inappropriate cross-reactivity was minimal. Samples were examined on an Olympus Provis AX module fluorescence microscope (objective: UPlanApo 40× 1.0 oil). The images were collected using a SPOT Insight Mono digital camera and were processed by Adobe Photoshop 7.0.

### ES cell differentiation into mesodermal, endothelial and smooth muscle cells

Differentiation of CCE cells first into Flk1^+ ^mesodermal cells and then into VE-cadherin^+ ^ECs and αSMA^+ ^SMCs that were able to form angiogenic sprouts was achieved following the protocol described in [13]. Micrographs were taken on an Olympus DP50 microscope and digital camera system. Images were processed using the Viewfinder Lite software and Adobe Photoshop 7.0.

Three dimensional collagen type I cultures were fixed with 3.5% paraformaldehyde for 1 h at room temperature. The samples were washed 3 × 15 min with PBS and were released from the wells and embedded in an 8% gelatin solution to provide rigidity. Fixation with paraformaldehyde was repeated for 1 h at room temperature. Excess gelatin was removed and samples were stored in 0.02% Sodium Azide in 1× PBS at 4°C. Sections of 200 μm thickness were prepared on a Micro-Cut H1200 vibrotome (Bio-Rad). Sections were permeabilised with 0.1% Triton X-100 for 1 h at room temperature, then washed 3 × 15 min with PBS. Primary antibodies against Pecam-1 and αSMA were added at appropriate concentrations and incubated overnight at 4°C. Sections were washed with PBS, then incubated in secondary antibody for 5 h at room temperature. After further extensive washing, sections were mounted onto slides using Gene Frame gaskets (Advanced Biotechnologies) and were sealed with coverslips. The samples were examined on a Leica DM-IRBE confocal microscope and images were processed using Leica TCS-NT and Adobe Photoshop 7.0 software.

### RNA extraction and RT-PCR

RNA extraction was performed with RNA-Bee (Tel-test, inc., Texas, US) and reverse transcription was performed using Superscript II (Invitrogen) according to the manufacturer's instructions. OligodT was used to initiate cDNA synthesis and the syndecan gene specific primers are as detailed in Table 2.

**Table 2:** Primers used in this study.

**Primer**	**Sequence**	**Purpose**
SDC1for	ATGAGACGCGCGGCGCTC	RTPCR
SDC1rev	GGCGTAGAACTCCTCCTGC	RTPCR
SDC2for	GCCTTGATGGCCTGTGTGTC	RTPCR
SDC2rev	GCATAAAACTCCTTAGTGGG	RTPCR
SDC3for	CGTGGCTGACGTAAGGACC	RTPCR
SDC3rev	CTCTAGTATGCTCTTCTGAA	RTPCR
SDC4for	CGAGAGACAGAGGTCATCGAC	RTPCR
SDC4rev	TGCGTAGAACTCATTGGTGG	RTPCR
bactfor	ATGGATGACGATATCGCTGCG	RTPCR
bactrev	CTAGAAGCATTTGCGGTGCAC	RTPCR
SDC2ecoRIfor	TATATAGAATTCGTAGGAGCCACATCCCTG	SDC2 cloning
SDC2ecoRIrev	TATATAGAATTCTTATGCATAAAACTCCTTAGTGG	SDC2 cloning
SDC4ecoRIfor	TATATAGAATTCGACTGGTTTGCGCTGTTGAA	SDC4 cloning
SDC4ecoRIrev	TATATAGAATTCTCATGCGTAGAACTCATTGGT	SDC4 cloning
IRESdelfor	GATATCCGCTAGCGCTACCGGACT	Deletion of CMV promoter from pIRES2-EGFP
IRESdelrev	ATGCATGGCGGTAATACGGT	Deletion of CMV promoter from pIRES2-EGFP
GFPfor	TATATAAAGCTTGCCACCATGGTGAGCAAG	GFP cloning
GFPrev	TATATAGGATCCATGATCTAGAGTCGCGGCC	GFP cloning

### Vectors and Cloning

The pEGFP-N1 plasmid was from Clontech. The pMC1neo PolyA plasmid was from Stratagene. The pCAGGS plasmid was a kind gift from Dr C. Huxley [17], Imperial College London. The pCAGIPuro vector was a kind gift from Dr H. Niwa, Riken Centre for Developmental Biology, Japan (Table 1).

Full length syndecan-2 and -4 cDNA obtained from RT-PCR of mouse brain was cloned into the EcoRI site of vector pCAGIPuro to create pCAGIPuro-sdc2 and pCAGIPuro-sdc4, respectively using the primers described in Table 2.

The GFP cDNA from pCAGGS was removed by EcoRI digestion, the product was gel purified then ligated to the EcoRI site of pCAGIPuro to create pCAGIPuro-GFP.

To insert the neomycin resistance cassette into the pCAGGS vector, pMC1neo polyA was digested with SalI and XhoI to release the neomycin resistance cassette, which was then ligated into the SalI site of pCAGGS to create pCAGGSneo-GFP.

For control purposes, the GFP cDNA was removed from pCAGGS by restriction digest with the enzyme EcoRI to create pCAGGS-ev. The neomycin resistance cassette from pMC1neo PolyA was then ligated to the SalI site of pCAGGS-ev to create pCAGGSneo-EV.

To create pIRES2-EGFP(β-actin), the EcoRI/SalI fragment of pLK444 [24] containing the β-actin promoter was gel purified and the overhangs were filled using Pfu polymerase. To remove the CMV promoter from pIRES2-EGFP (Clontech), PCR was performed using the primer pair IRESdelfor and IRESdelrev (Table 2). The primers were designed to yield a plasmid backbone of the pIRES2-EGFP containing an EcoRV site in place of the CMV promoter (pIRES2-EGFP-CMV). pIRES-EGFP-CMV was then cut with EcoRV, dephosphorylated and ligated to the blunt-ended β-actin fragment to generate pIRES2-EGFP(β-actin).

To create pLK444-GFP, the EGFP cDNA was amplified by PCR from pEGFP-N1 using the primer pair GFPfor and GFPrev (Table 2). The PCR product was digested with HindIII and BamHI and ligated to the HindIII/BamHI sites of pLK444 to yield pLK444-GFP.

### Transfection

For transfection in suspension, 150,000 CCE cells were plated per well of a six-well plate 24 h prior to transfection. Cells were trypsinised and washed in serum free DMEM. 1 μg DNA, 10 μl lipofectamine (Invitrogen) and serum free DMEM to a final volume of 50 μl were added to the cell pellet and incubated for 10 min at room temperature before transfer to a well of a 6-well plate containing 3 ml culture medium (supplemented with FCS). Fresh medium was added after 24 h. Fibroblasts were transfected with lipofectamine following standard procedures.

## Abbreviations

BSA, bovine serum albumin; CMV, cytomegalovirus; EDTA, ethylenediaminetetraacetic acid; FACS, fluorescence-activated cell sorting;GFP, green fluorescent protein; IRES, internal ribosomal entry site; LIF, leukemia inhibitory factor; PBS, phosphate buffered saline; PDGFR, platelet derived growth factor receptor; SSEA, stage specific embryonic antigen;VEGFA, vascular endothelial growth factor.

## Competing interests

The author(s) declare that they have no competing interests.

## Authors' contributions

ANA performed all of the experimental work presented in this paper. JRC and JRW both conceived of the study and were involved in drafting the manuscript. All of the authors have read and approved the final version of this manuscript.
